# Identification of significant m6A regulators and immune microenvironment characterization in ischemic stroke

**DOI:** 10.1038/s41598-024-53788-5

**Published:** 2024-02-11

**Authors:** Lili Zhao, Dingli Song, Tao Li, Ye li, Meijuan Dang, Qian Hao, Hong fan, Ziwei Lu, Jialiang Lu, Heyingwang, Xiaoya Wang, Yating Jian, Guilian Zhang

**Affiliations:** 1 Department of Neurology, The Second Affiliated Hospital of Xi’an Jiaotong University, No. 157 Xiwulu, Xi’an, 710004 China; 2 Department of Thoracic Surgery, The First Affiliated Hospital of Xi’an Jiaotong University, Xi’an, China; 3 Department of Oncology, The Second Affiliated Hospital of Xi’an Jiaotong University, Xi’an, 710004 Shaanxi China

**Keywords:** Genome informatics, Epigenetics, Diseases of the nervous system

## Abstract

The role of m6A modification in the regulation of the immune microenvironment (IME) of ischemic stroke (IS) is barely known. Thus, we aim to investigate the impact of m6A modification on the IME of IS and its diagnostic value in IS. We comprehensively assessed the m6A modification patterns, the relationship between these modification patterns and the characteristics of the IME. The m6A modification patterns of individual IS sample were quantified by m6Ascore. The performance of m6A phenotype-related genes as potential biomarkers was evaluated by the area under the receiver operating characteristic curve. Experimental validation was also performed by qRT-PCR. Six dysregulated m6A regulators were identified and a classification model consisting of four key m6A regulators (METLL3, RBMX, RBM15B, YTDHF3) could distinguish IS and healthy control samples well. METTL3 and YTHDF3 are closely related to circulating neutrophil abundance. Two distinct m6A modification patterns were determined which differed in immunocyte abundance. We also identified six m6A phenotype-related genes (*APOBEC3A*, *PTMA*, *FCGR3A*, *LOC440926*, *LOC649946*, and *FTH1L11*), and further explored their biological function. Among them, *APOBEC3A*, *FCGR3A*, and *FTH1L11* were positively associated with neutrophil abundance. *APOBEC3A* and *FCGR3A* were stable diagnostic m6A-associated genes in both the discovery and validation cohorts. This study reveals that m6A modification plays a non-negligible role in the formation of a diversified and complex IME in IS. The m6A phenotype-related genes could be diagnostic biomarkers of IS.

## Introduction

As we known, ischemic stroke (IS) is one of the leading causes of permanent disability and mortality worldwide^1^. The global IS burden and the economic costs associated with stroke are increasing^2^. Therefore, it is very important to identify novel biomarkers and elucidate the biological mechanisms of IS to improve the prevention and treatment of IS. Following IS attack, a series of harmful cascade events happen, including reactive oxygen species accumulation, immune cell infiltration, blood–brain barrier (BBB) breakdown as well as irreversible death of neurons. Besides, Epigenetic modifications including DNA methylation, histone acetylation, and non-coding RNA regulation are involved in complex, dynamic processes that modulate post-stroke gene expression, cellular injury response, motor function, and cognitive ability^3–6^. For example, Mayumi Asada et al.^7^ reported increased DNMT3a and 5mC levels in the core and peri-infarct region at 24 h following experimental cerebral ischemia. DNMT inhibition with RG108 treatment was shown to inhibit NMDR-induced neuron death, and decrease cerebral infarct volume. Additionally, several studies demonstrated that blood level of methylated DNA might serve as a biomarker for the diagnosis, prognosis, and treatment of stroke^8–10^. Consequently, understanding the role of epigenetic alterations in stroke may be beneficial to reveal the potential molecular mechanism and explore the innovative therapeutic target for IS.

N6-methyladenosine (m6A) modification is the most prevalent posttranscriptional internal mRNA modification^11^. And it forms when adenosine nucleotide acid is methylated at the nitrogen-6 position. The RNA m6A modification process is dynamically and reversibly regulated by three types of enzymes: m6A methyltransferases (“writers”), m6A demethylases (“erasers”), and m6A binding proteins (“readers”)^12^. A big writer complex, which consists of METTL3, METTL14, RBM15, RBM15B, WTAP, CBLL1, and ZC3H13, catalyze the installation of m6A^13^. This writer complex usually installs the m6A modification on a specific and consensus RNA sequence—RRACH (R = G or A; H = U, A or C) with 2–3 m6A-modified sites per transcript^14,15^. The two main erasers are FTO and ALKBH5^16,17^, which remove the m6A decoration from RNA by removing the adenosine^18^. Readers recognize the m6A decorations of target genes, which consist of YTHDC1, YTHDC2, YTHDF1, YTHDF2, YTHDF3, ELAVL1, IGFBP1, IGFBP2, IGFBP3, HNRNPA2B1, LRPPRC, HNRNPC, FMR1, and IGF2BP1^19^.

Previous research demonstrated that m6A modification is of critical importance in the regulation of vital biological processes and pathogenesis of a great many neurological diseases, such as Alzheimer’s disease (AD)^20,21^, Parkinson’s disease (PD)^22,23^ and gliomas^24^. Recent studies have demonstrated that abnormal m6A modifications are involved in ischemic cascade processes, e.g., oxidative stress^25^, apoptosis^26^, neurogenesis^27^, and inflammation^28^, indicating that m6A modifications are involved in the pathogenesis of IS. Lulu Zhu et al.^29^ provided direct evidence of increased global m6A levels in human peripheral blood by performing methylated RNA immunoprecipitation sequencing. However, basic researches mentioned above couldn’t provide understanding of the correlation between m6A methylation and the pathogenesis of IS from the macroscopic perspective. Besides, very limited studies that investigated the m6A modulator modifications patterns in human IS samples lacked of further validation and real-world verification. In addition, it was also interesting to identify novel biomarkers of IS from the aspect of m6A methylation. Therefore, we aimed to provide unique insight into the pathogenesis of IS by exploring the pattern of m6A modulator modifications, and evaluating the landscapes of immune infiltration during IS process. Moreover, we tried to identify valuable diagnostic biomarkers related to m6A regulators in human peripheral blood samples.

## Results

**Figure 1:**
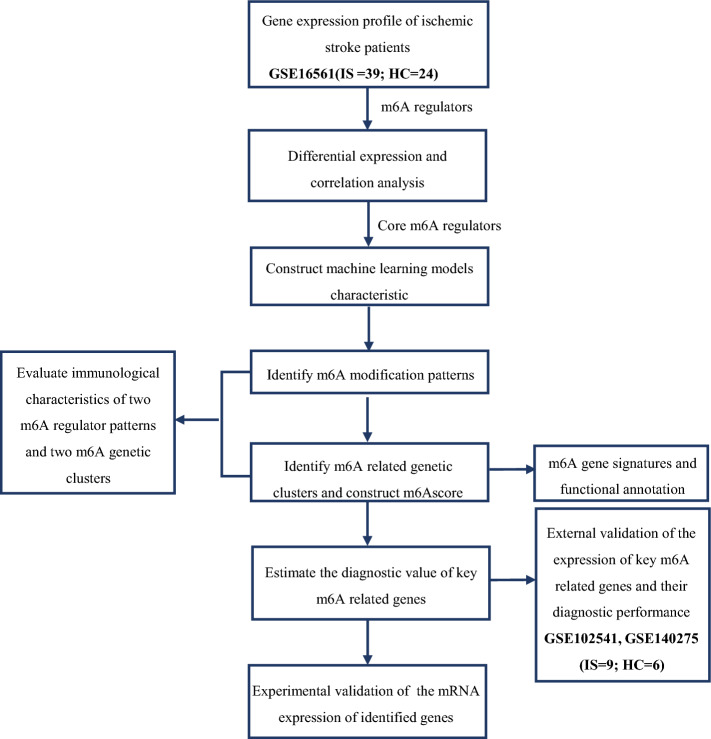
Flowchart of this study.

The workflow and data preprocessing of the overall study was showing in Fig. 1.

### The landscape of m6A regulators in IS

**Figure 2:**
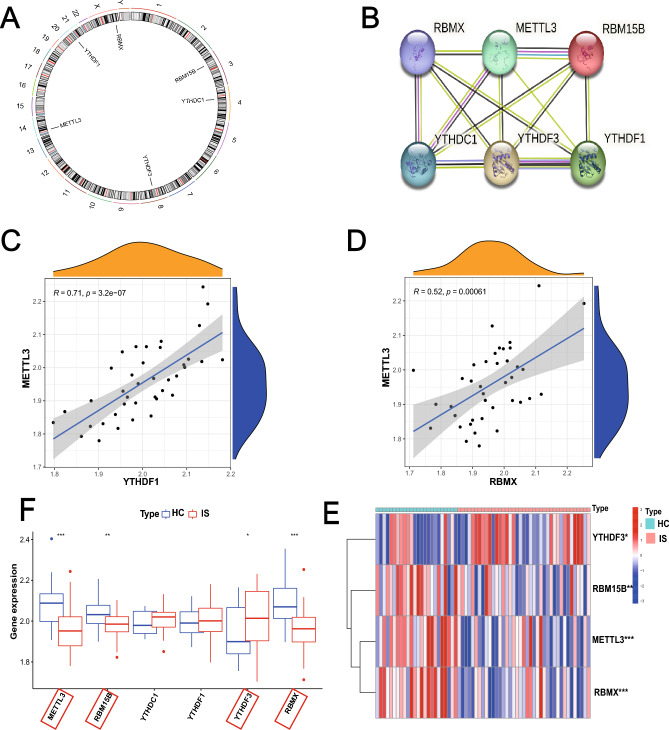
The landscape of m6A regulators in IS. (**A**) Position of the m6A regulators on 22 chromosomes from the GSE16561 cohorts. (**B**) Overview of the protein–protein interactions (PPI) across the 6 m6A RNA methylation regulators. (**C**, **D**) The two scatter plots indicate the three most correlated m6A regulators: METTL3, YTHDF1 and RBMX. (**E**) Heatmap of significantly different in the expression levels of 4 regulators in IS and HC samples (Healthy control; HC). (**F**) Box plot to show the expression level of 6 m6A regulators in IS and normal samples (*p < 0.05; ** p < 0.01; *** p < 0.001).

Twenty-six m6A regulators were investigated at first, but only six m6A regulators were extracted in the training dataset, which included 3 readers (YTHDC1, YTHDF3, YTHDF1), and 3 writers (RBMX, METTL3, RBM15B). Figure 2A displayed the location of the six differential-expressed m6A regulators on chromosomes. This information could reveal the interaction between genes and help us to find loci might be modified by m6A regulators. A PPI network that described the regulatory interactions among these m6A regulators was shown in Fig. 2B. Subsequently, we explored the correlated expression of different regulators in the whole sample (Fig. 2C,D), where special attention was paid to the correlation between writers and readers. METTL3 and YTHDF1 (r = 0.71), METTL3 and RBMX (r = 0.52) presented remarkably positive correlation in expression. Three writers showed significantly decreased expression in IS compared to healthy controls (HC) including METTL3, RBM15B, RBMX. And one reader, YTHDF3, showed increased expression in IS while the other two readers including YTHDC1, YTHDF1 were not differently expressed (Fig. 2E,F).

### m6A regulators as potential biomarkers of IS

**Figure 3:**
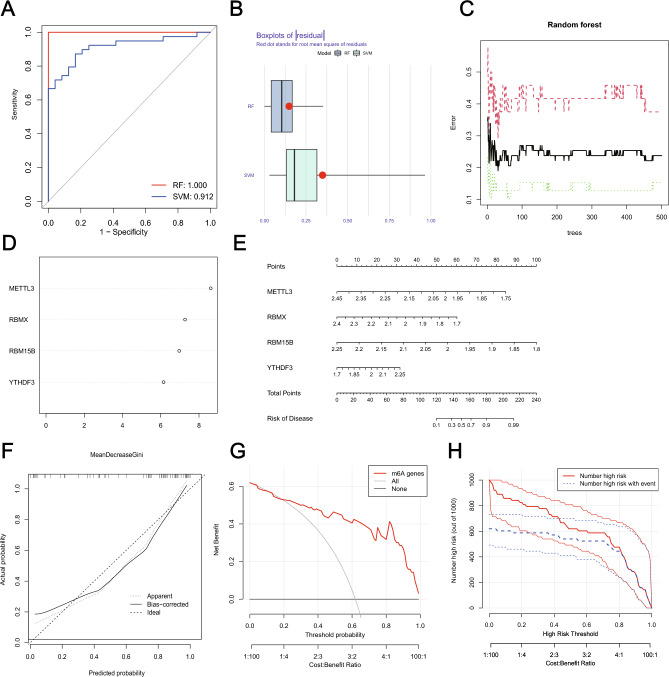
m6A regulators as potential biomarkers of IS. (**A**) ROC curve of Support vector machine (SVM) model and random forest (RF) model. The discrimination ability for healthy and AIS cases by m6A regulators was analyzed by ROC curve and evaluates by AUC value. (**B**) Box plots of residuals in SVM and RF. (**C**) Error graph of the RF model. (**D**) The importance score of four m6A differential genes. (**E**) Nomograms estimating the risk scores of four m6A regulators related to IS. (**F**) Calibration plots of the model to evaluate the uniformity between anticipated and IS. (**G**) Decision curves for two AIS-specific risk predictive models. The clinical impact curves of the models are displayed in (**H**).

To investigate the contribution of m6A regulators to IS pathogenesis, we developed SVM and RF models to identify candidate m6A regulators to anticipate the onset of the IS. The ROC curve showed that both RF and SVM models had good diagnostic performance in classifying normal and IS samples (Fig. 3A). Nonetheless, we chose the RF model as the best fit due to the residual box line plot demonstrated that the RF model had the smallest residuals (Fig. 3B,C). As was shown in Fig. 3D, four core m6A regulators had importance scores > 2. Finally, using these four core m6A regulators as building blocks, a nomogram score was built to show the contribution of each m6A modulator to the risk of IS (Fig. 3E). The calibration curves demonstrated that the predictions of the nomogram were correct (Fig. 3F). The nomogram model performed well in distinguishing IS patients from HC, which was proved by the fact that the red line in the DCA curve was far away from the gray line (Fig. 3G). The clinical impact curve showed significant predictive performance of the nomograph model (Fig. 3H). The above analyses indicated that the expression imbalance of m6A regulators plays vital role in IS occurrence.

### m6A regulators are associated with immune responses in IS

**Figure 4:**
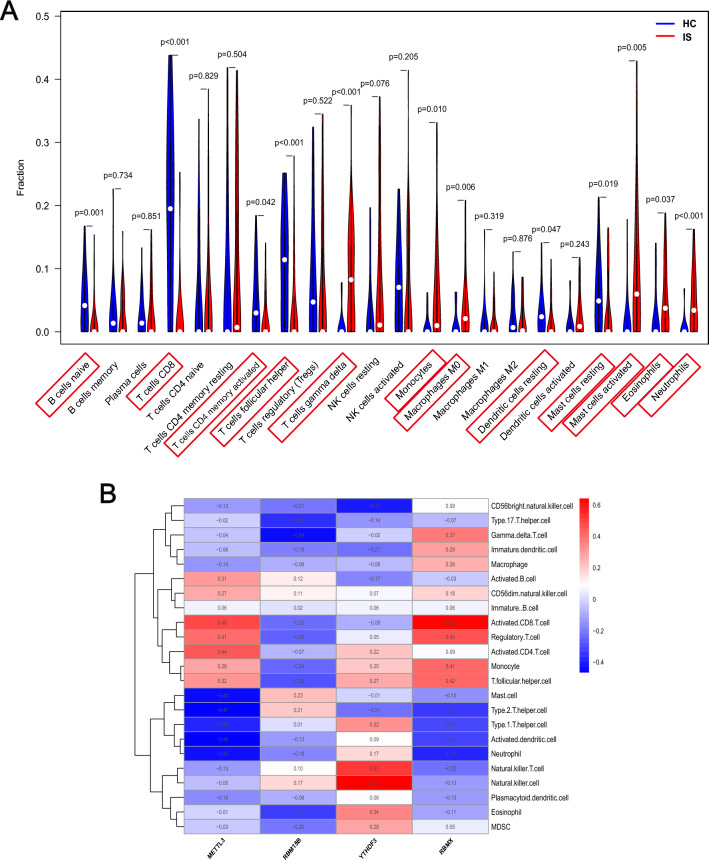
m6A regulators are associated with immune responses in IS. (**A**) The violin plot shows the differences in the abundance of 22 immunocytes in HC and IS samples. (**B**) The correlation between immunocytes and m6A regulators.

To investigate the association between the immunological microenvironment (IME) and m6A regulators in IS, we first analyzed the differences in peripheral immune cells in HC and IS samples. The results revealed that there was a lower abundance of T cells and B cells, meanwhile a higher abundance of myeloid cells like neutrophils, and eosinophils in IS samples (Fig. 4A). Moreover, we wanted to find out the association between the expression of the above four key regulators (METTL3, RBM15B, YTHDF3, RBMX) and each immune cell type. According to the results of correlation analysis, four key regulators were closely correlated with several kinds of immune cells in IS samples (Fig. 4B). For example, neutrophil has a negative correlation with METTL3 (r =  − 0.43) and a positive correlated with YTHDF3 (r = 0.17), indicating that low expression of METTL3 and high expression of YTHDF3 was correlated with increased neutrophil abundance in IS. All results above demonstrated that the key m6A modulators were closely related to the IME formation in IS.

### m6A methylation modification patterns mediated by four regulators

**Figure 5:**
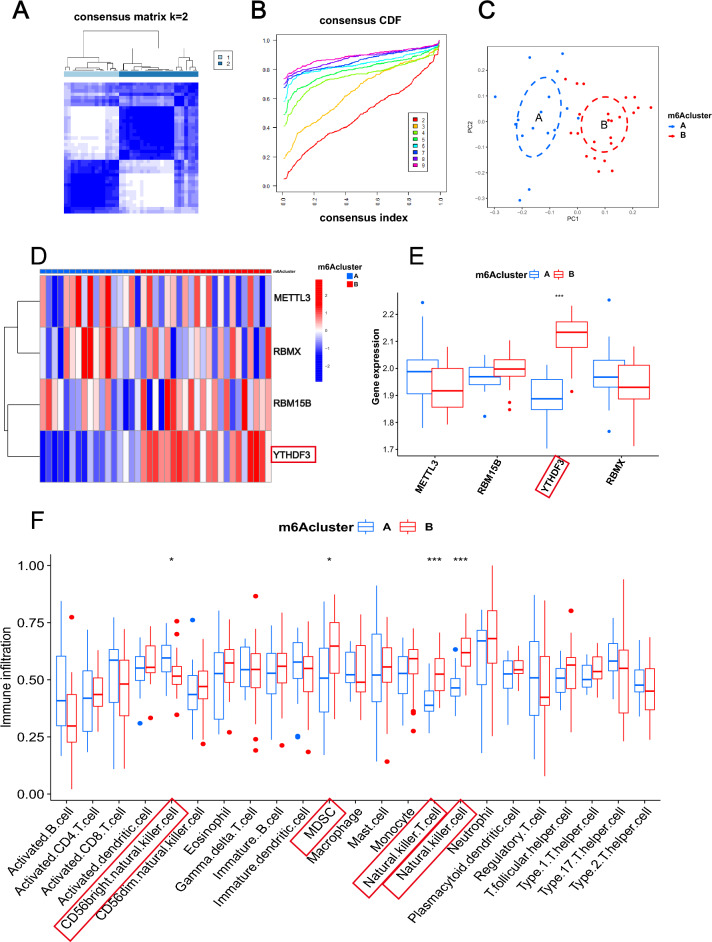
m6A methylation modification patterns mediated by four regulators. (**A**) Heatmap of the matrix of co‐occurrence proportions for IS samples. (**B**) Consensus clustering cumulative distribution function (CDF) for k = 2–9. (**C**) Principal component analysis for the transcriptome profiles of two m6A subtypes, showing a remarkable difference on transcriptome between different modification patterns. (**D**, **E**) The expression status of 4 m6A regulators in the two m6A subtypes. (**F**) The immune cells characterization in 2 m6A modification patterns (*p < 0.05; **p < 0.01; ***p < 0.001).

To explore the m6A modification patterns in IS, unsupervised consensus clustering analysis was conducted for IS samples according to the expression of four key m6A regulators identified above (Fig. 5A,B). Two distinct subtypes of IS were identified with quantitatively different expressions of four m6A regulators, including 16 samples in subtype‐1, and 23 samples in subtype‐2 (Fig. 5C). We termed these patterns as m6Acluster A/B, respectively (Supplementary Table S1). The expression of RBM15B and YTHDF3 were higher in m6Acluster B. While METLL3 and RBMX expressed lower in m6Acluster B. But only the expression of YTHDF3 was significantly different between two m6Aclusters (Fig. 5D,E). Then we compare the component differences of immune cells among the two m6A modification patterns to determine the immune cell type in IS. Generally, m6Acluster B was relatively enriched in the innate immune cell compared with m6Acluster A. Specifically, m6Acluster B had more CD45 brighter natural killer cells, MDSC, natural killer T cells, and natural killer cells (Fig. 5F). These results suggested m6Acluster B mediates an active immune response, while m6Acluster A leads to a mild immune response in IS. The above results once again demonstrated that m6A methylation modification played a crucial regulatory role in shaping distinct IME in IS.

### Generation of m6A regulators related signatures and functional annotation

**Figure 6:**
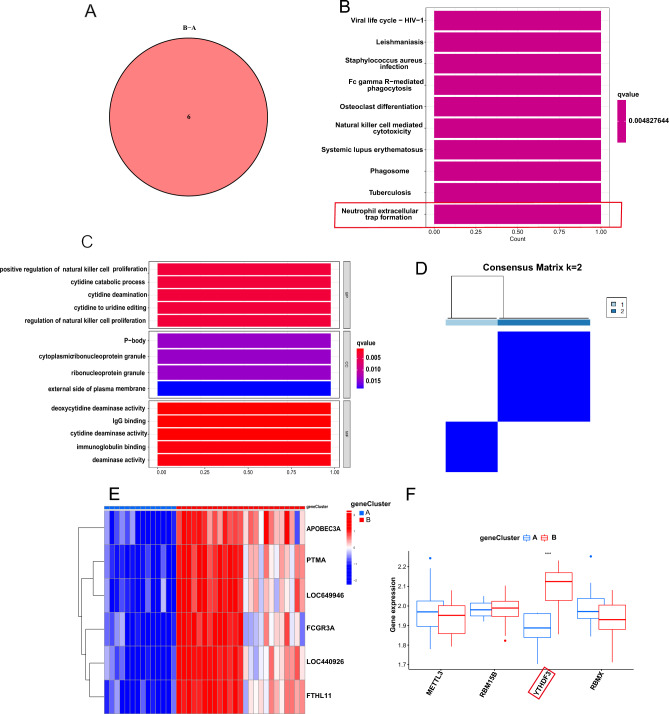
Generation of m6A gene signatures and functional annotation. (**A**) Six m6A phenotype-related genes shown in Venn diagram. (**B**) KEGG pathway (**C**) and GO function enrichment analysis revealed the biological characteristics of m6A phenotype-related genes. (**D**) Unsupervised clustering of 6 genes in two gene patterns. (**E**) Matrix of co-occurrence percentages for IS samples visualized as a heatmap. (**F**) Expression of four distinct m6A regulators in each of the two gene subsets.

To further investigate the potential biological behavior of each m6A modification pattern, we determined six m6A phenotype-related DEGs (APOBEC3A, PTMA, FCGR3A, LOC440925, FTH1L11) using the “limma” package (Fig. 6A). Subsequent KEGG pathway enrichment analysis revealed their involvement in neutrophil extracellular traps formation, phagosome, and natural killer cell-mediated cytotoxicity (Fig. 6B). GO enrichment analysis indicated that they were involved in processes like regulation of immunocyte proliferation (Fig. 6C), which confirmed again that m6A modification played a nonnegligible role in the immune regulation in IS microenvironment. Then we performed an unsupervised cluster analysis based on the six m6A phenotype-associated genes to classify IS patients into different genomic subgroups. Consistent with the m6A modification patterns, the unsupervised cluster algorithm also determined two different m6A modification genomic phenotypes, named geneClusters A/B (Fig. 6D, Supplementary Table S2). In these two clusters, the expression of YTHDF3 was significantly high in geneCluster B (Fig. 6E,F), which was consistent with the predicted results of the m6A methylated modification patterns.

**Figure 7:**
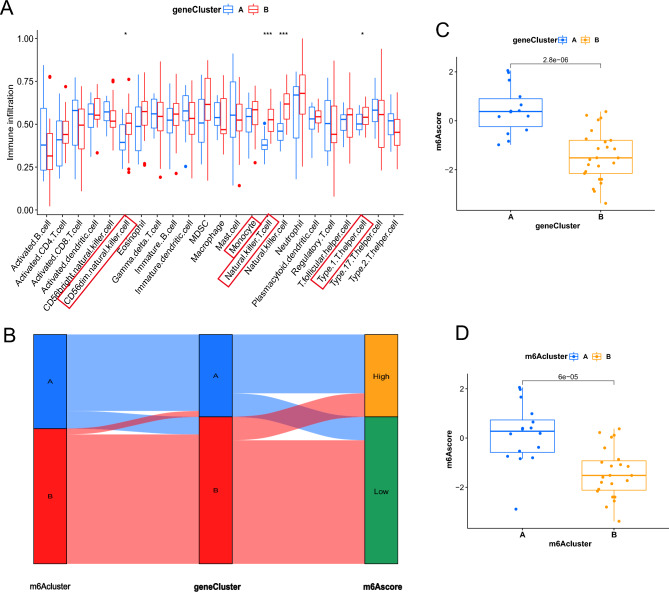
Characteristics of m6A-related phenotypes. (**A**) The abundance differences of each immunocyte type in 2 genetic clusters. (**B**) Alluvial illustration representing the variation in m6A clusters, genetic clustering, m6A score. (**C**) Differences in m6A score among the two genetic clusters (*p* < 0.001, Kruskal–Wallis test). (**D**) Differences in m6A score among the two m6A modification patterns (*p* < 0.001, Kruskal–Wallis test), (*p < 0.05; **p < 0.01; ***p < 0.001*p < 0.05; **p < 0.01; ***p < 0.001).

To better characterize m6A-associated gene patterns, we compared the differences in peripheral immune cell abundance between two genetic patterns (Fig. 7A). Similar to m6A patterns, geneCluster B displayed higher immune cell abundance compared to geneCluster A. The geneCluster B also had more CD45 brighter natural killer cells, and natural killer T cells. Thus, we thought that the significant differences in m6A gene expression across the two genetic clusters might contribute to the formation of distinct IME. Despite all this, these assays were only based on IS populations and cannot precisely predicted the m6A modification patterns of individual patient. Considering the individual heterogeneity and complexity of m6A modification, we constructed a set of scoring systems, m6Ascore, to quantify the m6A modification pattern of individual patient with IS based on these phenotype-related genes. The alluvial diagram was used to visualize the attribute changes of individual patients (Fig. 7B). The Kruskal–Wallis test revealed a significant difference in m6Ascore between m6A gene clusters. m6Acluster A corresponds to geneCluster A displaying a high m6A score, while m6Acluster B correspond to geneCluster B having a lower m6A score. Besides, geneCluster A has a higher m6A score, whereas geneCluster B displayed a lower m6A score, indicating that a low m6A score might have a strong relation with increased immune cells (Fig. 7C). In addition, the m6A score was significantly higher in m6Acluster A and lower in m6Acluster B (Fig. 7D). These results suggested that low m6A scores are negatively correlated with immune stimulation. The m6A score might be an effective indicator to evaluate the m6A modification pattern of individual IS patient and further assess the immune cell characteristics of IS.

### Diagnostic performance of m6A phenotype‐related genes in IS

**Figure 8:**
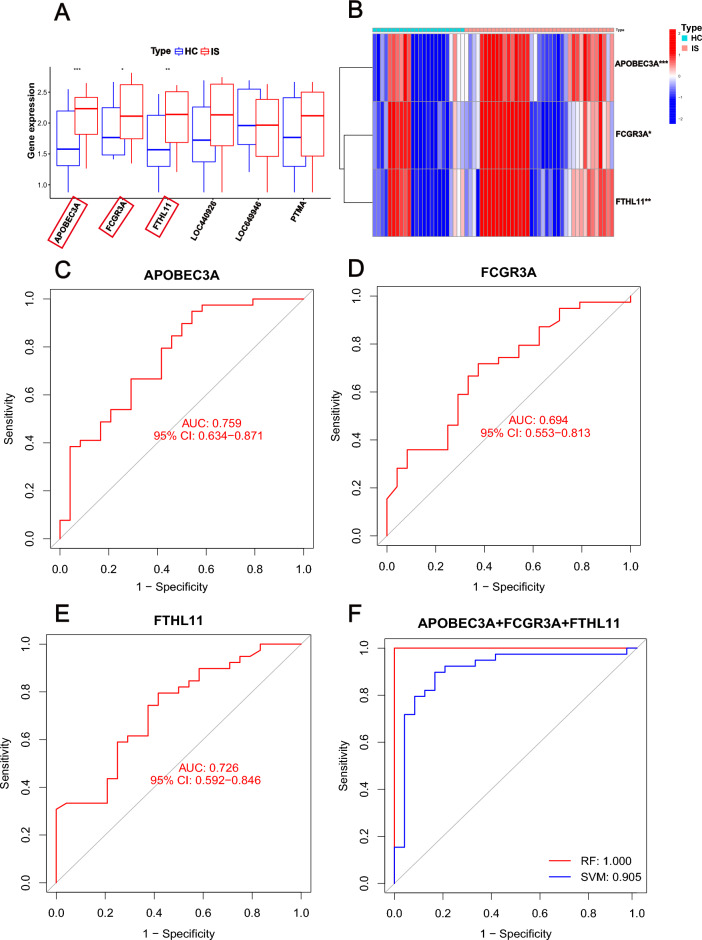
Diagnostic performance of m6A phenotype‐related genes in IS. (**A**) Bar plot of the expression of six m6A modulator associated genes in HC and IS patients. (**B**) Heatmap of three differential m6A modulator associated genes in HC and IS patients. (**C**–**F**) ROC of APOBEC3A, FCGR3A, FTH1L11, and APOBEC3A + FCGR3A + FTH1L11 (*p < 0.05; **p < 0.01; ***p < 0.001).

To explore the diagnostic performance of m6A phenotype‐related genes in IS, we first explore the expression of the six genes in HC and IS patients. The expressions of APOBEC3A, FCGR3A, and FTH1L11 were significantly increased in IS patients (Fig. 8A,B). Then the diagnostic performances of the three genes to distinguish patients with IS and HC were appraised via ROC analysis in this cohort. The AUC was 0.759 (95% CI = 0.634–0.871) for APOBEC3A (Fig. 8C), 0.694 (95% CI = 0.553–0.813) for FCGR3A (Fig. 8D), and 0.726 (95% CI = 0.592–0.846) for FTH1L11 (Fig. 8E). The AUC of ROC was improved when combined these three genes (AUC_RF_ = 1.00; AUC_SVM_ = 0905) (Fig. 8F). These results indicated that m6A phenotype-related genes performed well in classifying HC and IS people, further indicating that m6A regulators were indeed important in IS development.

### m6A phenotype-related genes take part in neutrophil activation

**Figure 9:**
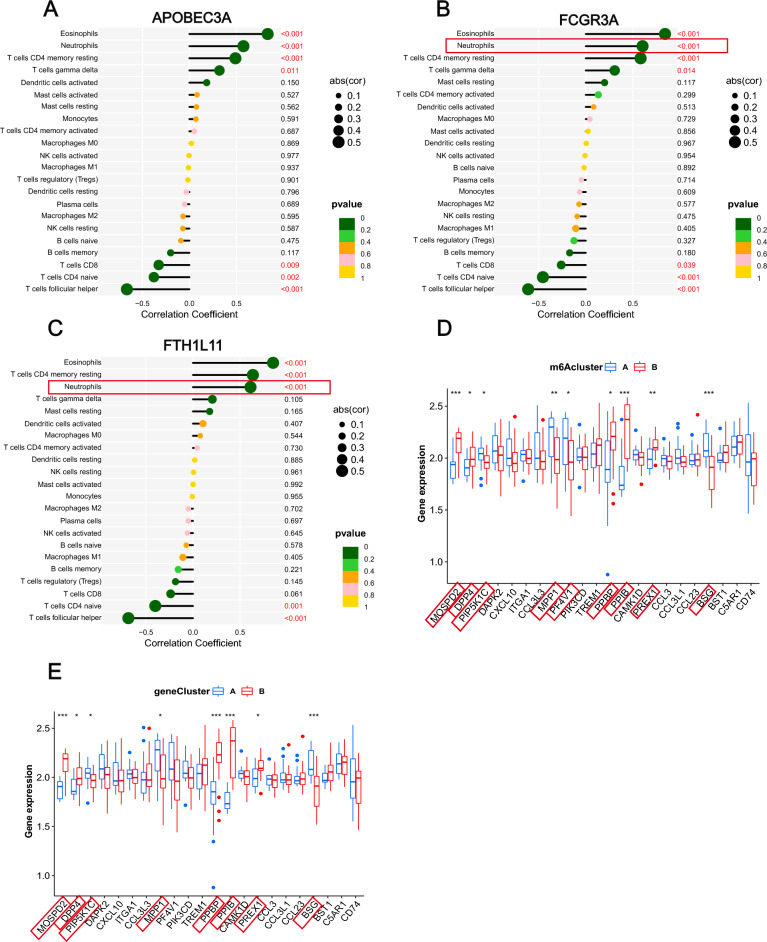
m6A phenotype-related genes take part in neutrophil activation. Correlation analysis between m6A modulation related genes, APOBEC3A (**A**), FCGR3A (**B**), FTH1L11 (**C**) and immune cells were visualized by Lollipop. (**D**) The expression of neutrophil chemotaxis in the two m6A subtypes. (**E**) The expression of neutrophil chemotaxis in the two m6A regulator related genetic clusters (*p < 0.05; **p < 0.01; ***p < 0.001).

To understand the roles of APOBEC3A/FCGR3A/FTH1L11 in the IME formation in IS, we investigated immune cell landscapes and their relationship with APOBEC3A/FCGR3A/FTH1L11. We found that all of these three genes were positively associated with eosinophils, neutrophils, T cells CD4 memory resting (*p* < 0.001) (Fig. 9A–C). As we know, the number of peripheral blood neutrophils increased soon after stroke onset. Besides, a high neutrophil count indicated poor stroke outcomes^30^. So, we aimed to figure out whether genes related to neutrophil chemotaxis were different between various m6A clusters and different genetic clusters. The results indicated that most of the neutrophil chemotaxis increased in both m6Acluster B and geneCluster B, such as MOSPD2, DPP4, PPBP, PPIB, and PREX1, which was consistent with more peripheral neutrophils (Fig. 9D,E). Thereby, we speculated that IS patients divided into m6Acluster B and geneCluster B may have a worse prognosis when compared with IS patients in m6Acluster A and geneCluster A.

### Validation the diagnostic performance of m6A phenotype‐related genes

**Figure 10:**
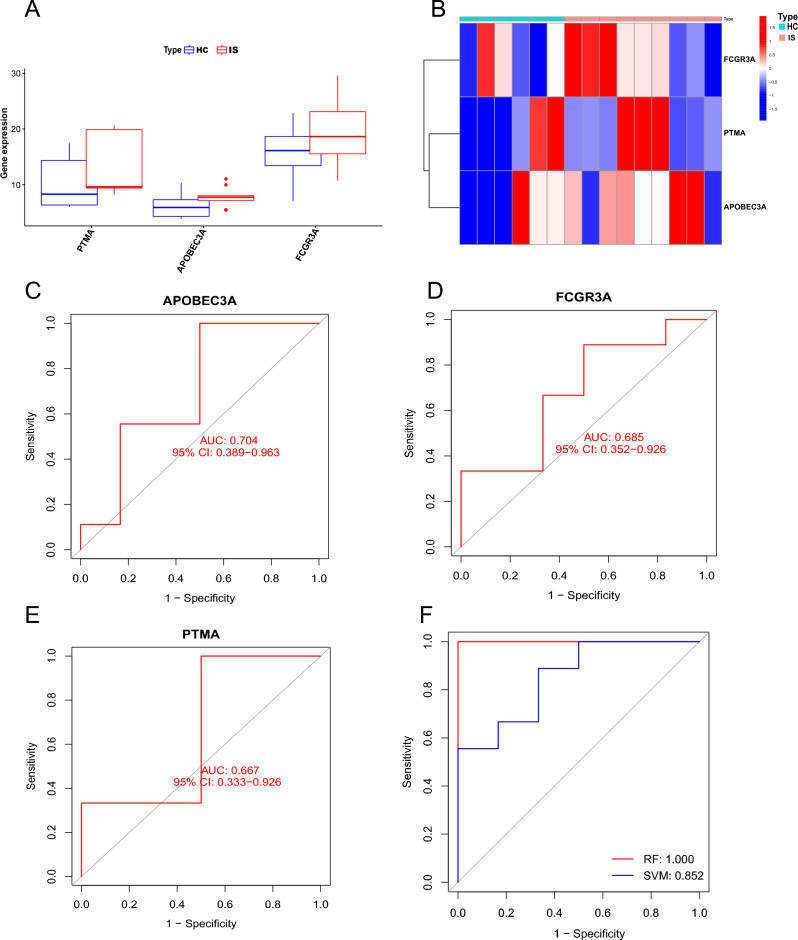
Validation the diagnostic performance of m6A phenotype‐related genes. (**A**) Bar plot of the expression of three m6A modulator associated genes in validation cohort. (**B**) Heatmap of three differential m6A modulator associated genes in validation cohort. (**C**–**F**) ROC of APOBEC3A, FCGR3A, FTH1L11, and APOBEC3A + FCGR3A (*p < 0.05; **p < 0.01; ***p < 0.001).

To validate our results, we utilized another two independent, human, peripheral blood datasets, GSE102541 and GSE140275, as verification. Three m6A phenotype-related genes were found in our validation cohort with similar expression tendency in explore cohort. As is shown in Fig. 10A,B, PTMA, APOBEC3A, and FCGR3A were relatively highly expressed in IS patients. Given the limited sample size of only 15 samples in this cohort, no significant statistical difference was found between HC and IS patients. Then ROC was used to estimate the diagnostic capability of PTMA, APOBEC3A, and FCGR3A in the validation dataset. The AUC was 0.704 (95% CI = 0.389–0.963) for APOBEC3A (Fig. 10C), 0.685 (95% CI = 0.362–0.926) for FCGR3A (Fig. 10D), and 0.667 (95% CI = 0.333–0.926) for PTMA (Fig. 10E). The diagnostic performance of APOBEC3A combined with FCGR3A for the diagnosis of IS also proved that m6A phenotype-associated genes could distinguish between IS patients and HC people (AUC_RF_ = 1.00; AUC_SVM_ = 0.852) (Fig. 10F).

### Validation of the expression levels of key m6A regulators and m6A phenotype‐related genes in IS

**Figure 11:**
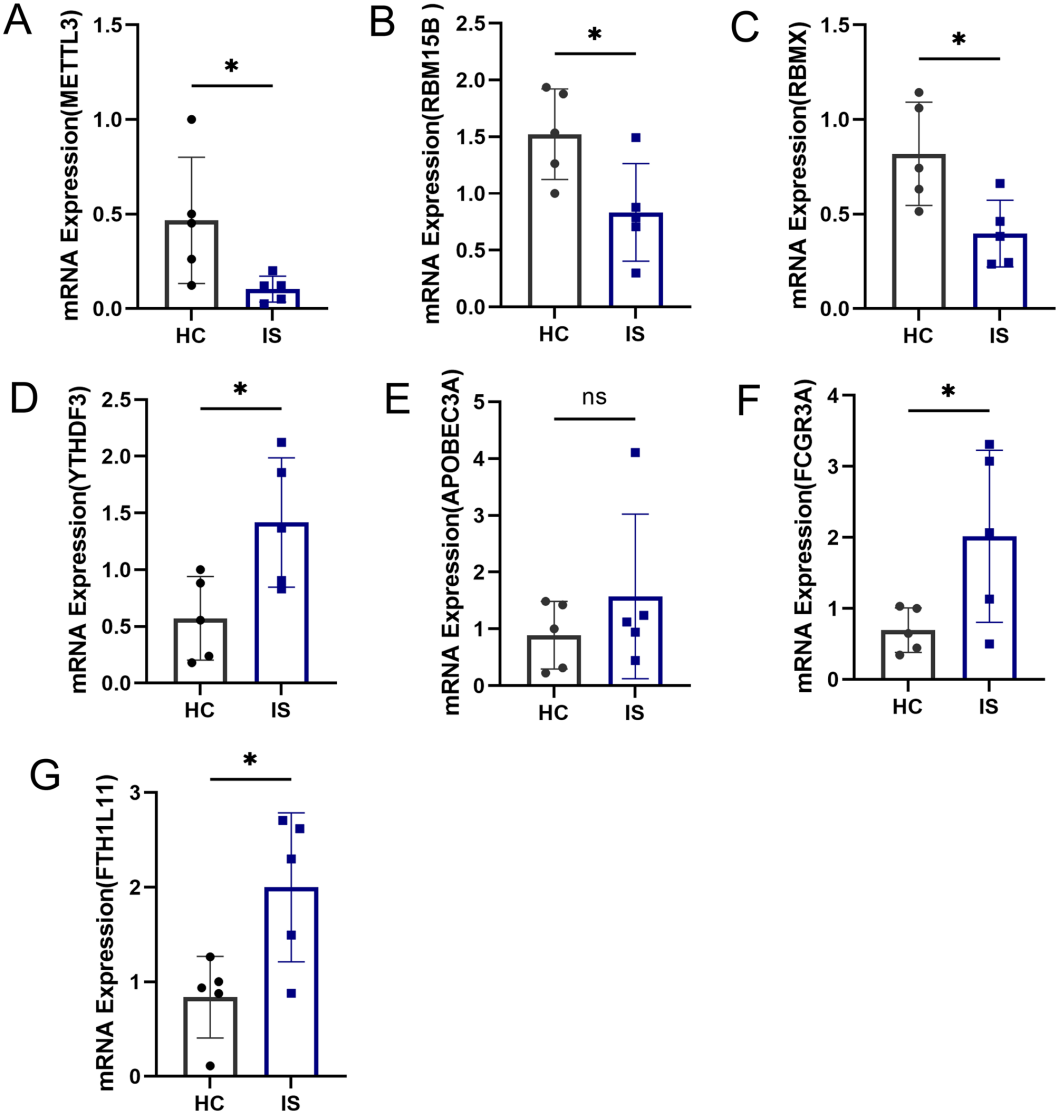
Validation of the expression levels of key m6A regulators and m6A phenotype‐related genes in IS. (**A**–**D**) mRNA expression of four key m6A regulators; (**E**–**G**) mRNA expression of three m6A phenotype-related genes (*p < 0.05; **p < 0.01; ***p < 0.001).

To further verify the expression of these identified key m6A regulators and m6A phenotype-related genes in IS, five pairs of HC and IS peripheral blood samples were used to detect the mRNA expression level of these genes by qRT-PCR (Supplementary Table S3). As shown in Fig. 11A–D, METTL3, RBMX, and RBM15B were significantly downregulated in IS, while YTHDF3 was upregulated in IS compared to the levels in HC. Besides, the mRNA expression of three diagnostic m6A phenotype-related genes was upregulated in IS (Fig. 11E–G). These results were consistent with our findings of bioinformatic analysis.

## Discussion

Stroke occurs when a cerebral artery was occluded and blood flow interrupted and characterizes by complex mechanisms of innate and adaptive immune cell-mediated inflammatory injury^31^. So far, a number of published studies explored the role of m6A on tumor microenvironment, and the results confirmed its fundamental role in tumor immunity^32,33^. Thus, we assumed that m6A modification may produce a similar effect in shaping the IME in IS. Here, we systematically investigated the m6A modification patterns, explored potential diagnostic biomarkers, and validated our results by qRT-PCR.

And we believe this will contribute to sharpening our understanding of immune response in IS, and guiding more effective immunotherapy strategies.

A series of analyses was conducted to reveal how m6A could shape the immune reactions in IS as well as enrich immunocytes, and the following findings were discovered. Firstly, given the limited high-quality datasets and limited sample size for each dataset, only six m6A regulators were identified in our study. Among them, the expression of four m6A regulators was significantly different between HC and IS people. A classifier established by m6A regulators performs well in distinguishing HC and IS samples, which proved the vital role of m6A regulators in IS again. METTL3, RBM15B, YTHDF3, and RBMX may be the most important ones among the six m6A regulators because of their large fold change of expression and high importance score. Recent study demonstrated elevated global m6A levels in the peripheral blood of patients with IS by performing Methylated RNA immunoprecipitation sequencing (MeRIP-seq)^34^. The overall level of m6A in IS was increased, indicating that the expression of most m6A methyltransferases (writers) in IS should be increased, while the expression of demethylases (erasers) should be decreased. Interestingly, our results showed generally increased expression of YTHDF3 and decreased expression of METTL3, RBM15B, RBMX (Fig. 2A) which seemed unreasonable. However, our results were not contradictory to previous studies. For example, several previous studies have demonstrated m6A demethylase FTO declined significantly after cerebral ischemia^35–38^. This was one important factor contributing to increased m6A methylation level. Most studies on METTL3 (writer) in IS also indicated decreased mRNA and protein expression after IS^39,40^, which was similar to that of our results. Besides, published article on m6A readers mainly focused on YTHDF1, YTHDC1. Both of them were upregulated after cerebral ischemia^41–43^. Moreover, the expression levels of m6A regulators might reflect specific dynamic balance on this time point. Some post-transcriptional modification or post-transcriptional regulatory mechanisms might promote the expression of m6A readers and inhibit the expression of m6A writers. In addition, there were upstream regulators that could influence the expression of m6A readers and writers.

Secondly, we explored the association between m6A regulators and peripheral immunocytes of IS. We found the four key m6A regulators are closely correlated to immunocytes, implying that m6A modification played an essential role in IS IME regulation. Besides, circulating neutrophil abundance had a negative correlation with METTL3 and a positive correlation with YTHDF3. Neutrophils are of vital importance to innate immunity in IS. Activation of circulating neutrophils contributed to thrombosis and the “no perfusion” phenomenon which account for neurological function deterioration^44,45^. Recent studies provided support on our findings. One research demonstrated that the levels of a series of inflammatory cytokines, including IL-8, were elevated when METTL3 was inhibited^46^. A previous study reported that IL-8 could attract neutrophils to inflammatory foci^47^. And the research did observe increased CD45^+^CD11b^+^Ly6G^+^ cells (neutrophils) in the tumors after knockdown of METTL3 in BCPAP cells^47^. Liu et al.^47^ found that the expression of YTHDF3 was positively related to the infiltration of neutrophils in hepatocellular carcinoma. These findings may provide a revealing insight into the immune regulation mechanism of m6A modification in IS.

Thirdly, based on four key m6A regulators, two distinct m6A methylation modification patterns with unique immune characteristics were identified. The modification of m6Acluster B had more circulating immunocytes and more active immune reactions compared with m6Acluster A. Given the immune characteristics of each subtype, it confirmed the reliability of our classification of immune phenotypes for different m6A regulators. Many oncology diseases were subtyped according to their molecular characteristics. And the identification of novel molecular subtypes was beneficial to make a more effective treatment strategy^48,49^. In 2019, Marios K. Georgakis et al.^50^ found genetic predisposition to higher circulating levels of monocyte chemoattractant protein-1 was associated with higher risk of stroke. Besides, associations were also found with etiologic stroke subtypes, specifically large-artery stroke and cardioembolic stroke. Therefore, we thought the two distinct m6A modification pattern subtypes of peripheral blood might be an alternative classification of IS.

Further, in this study, we demonstrated that the mRNA transcriptome differences between distinct m6A modification patterns was significantly associated with immune related biological pathways. These DEGs were considered as m6A-related signature genes. Similar to the clustering results of the m6A modification phenotypes, two genomic subtypes were identified based on m6A signature genes, which were also significantly correlated with immune activation. Specifically, KEGG pathway enrichment analysis showed these genes involving in neutrophil extracellular traps formation, phagosome, natural killer cell mediated cytotoxicity. Furthermore, geneCluster B had more CD45 brighter natural killer cell, natural killer T cell. YTHDF3 was also up-regulated in geneCluster B. This demonstrated again that the m6A modification was of great significance in shaping different IS IME landscapes. Based on the DEGs, a scoring system, m6A score, was established to evaluate the m6A modification pattern of individual patient with IS. The m6A modification pattern characterized by immune-active phenotype exhibited a lower m6Ascore, while the pattern characterized by immune-inhibiting phenotype showed a higher m6Ascore. This suggested m6Ascore was a reliable and robust tool for comprehensive assessment of individual IS m6A modification patterns, which could be used to further determine the IS immune phenotypes. However, we couldn’t further identify the performance of m6A score as a prognostic biomarker and its’ association with clinical characteristics in IS due to the lack of clinical data. Thus, further researches are still required.

Last but not least, to expand the possibilities of a molecular diagnosis for IS, we tested whether the three m6A phenotype-related genes (APOBE3CA, FCGR3A, FTH1L11) could potentially serve as circulating diagnostic biomarkers of IS. All the genes showed a good capability to classify HC and IS people in the training set. Though only APOBE3CA, FCGR3A, and PTM were discovered in the validation cohort, the combination of APOBE3CA with FCG3A still had high diagnostic values. Of the two, APOBEC3A was first identified as a biomarker in IS, while FCGR3A was previously discovered in human cerebral infarction samples^51^. FCGR3A was also considered as a new therapeutic option for IS in previous research^52^, which powerfully proved the results of this study.

Generally, our results provide a new perspective on IS pathogenesis research from the m6A modification mechanism and identified m6A related circulating diagnostic biomarkers of IS. However, there are also some limitations in our study. Above all, this study is based on bioinformatics analysis, and many results are not verified by experiments. But there are numerous studies based on GEO data analysis, and we believed that our results are reliable. Besides, clinical characteristics were unavailable to us and this limited us to further reveal the association of m6A methylation with clinical features of IS patients and the impact of m6A modification on prognosis of IS. And this reminds us of the importance of collecting clinical characteristics when sequencing samples. Besides, our external validation dataset has a limited sample size. Nonetheless, similar m6A regulator-related genes were found between healthy and IS samples in the validation data set. And this indicated that our findings were stable.

## Materials and methods

### Data preprocess

Three RNA-seq datasets GSE16561 (n = 63)^53^, GSE102541 (n = 9)^54^, and GSE140275^55^ (n = 6) were downloaded from the Gene Expression Omnibus (GEO) database (https://www.ncbi.nlm.nih.gov/gds/). GSE16561 was used as the training set which included 39 cases of IS samples and 24 of normal samples. Moreover, the GSE102541 and GSE140275 datasets were used as the validation sets. These two datasets included 9 cases of IS samples and 6 cases of normal samples. The above samples were all taken from human peripheral blood. Firstly, Gene probes were annotated as gene symbols using Perl script. Secondly, Media value was calculated and used as the gene expression value of the duplicate gene symbol, which was just greater than 0. Then the expression value was pre-processed by the “Normalize Between Arrays” function in the “limma” R package (bioconductor.org/packages/release/bioc/html/limma.html). As the two validation datasets, the “sva” package in R was used to merge and normalize the raw data of GSE102541 and GSE140275 after annotation and duplication. After that, data preprocess was completed. The relevant m6A regulators investigated in this study were collected from previous literature^56,57^ and listed in Supplementary Table S4.

### Expression landscape of m6A regulators between different samples and correlation analysis

First, we used the “limma” R package to identify differentially expressed m6A regulators between IS and control samples. The *p* < 0.05 was selected as the cut-off threshold. And four core m6A regulators were identified. The heatmaps and bar plots were executed to visualize the difference by “pheatmap” (https://CRAN.R-project.org/package=pheatmap), “reshape2”^58^ and “ggpubr” R packages (https://CRAN.R-project.org/package=ggpubr), respectively. Spearman correlation analysis was employed to assess the expression relationships among differentially expressed m6A regulators using “limma” and visualized by “ggplot2”^59^, “ggExtra” (https://CRAN.R-project.org/package=ggExtra), and “ggpubr” R packages, focusing on the correlation between readers and writers. And the significant correlation criteria were set at correlation coefficient > 0.5, *p*-value < 0.001. The protein–protein interaction among these m6A regulators was explored using STRING (http://string.embl.de/).

### Screening of key m6A regulators

The support vector machine (SVM) and random forest (RF) models were performed to predict the diagnosis of IS. Four key m6A regulators identified above were subjected to SVM and RF using “caret”^60^, “kernlab” (https://CRAN.R-project.org/package=kernlab) and “randomForest” (The R Journal: Classification and regression by randomForest (r-project.org) R packages.

The codes and parameters of SVM were as follows:

- ksvm(x, y = NULL, type = NULL,
- kernel = "stringdot", kpar = list(length = 4, lambda = 0.5),
- C = 1, nu = 0.2, epsilon = 0.1, prob.model = FALSE,
- class.weights = NULL, cross = 0, fit = TRUE, cache = 40,
- tol = 0.001, shrinking = TRUE, …,
- na.action = na.omit)

The codes and parameters of RF were list below.

- randomForest(x, y = NULL, xtest = NULL, ytest = NULL, ntree = 500,
  - mtry = if (!is.null(y) && !is.factor(y))
  - max(floor(ncol(x)/3), 1) else floor(sqrt(ncol(x))),
  - weights = NULL,
  - replace = TRUE, classwt = NULL, cutoff, strata,
  - sampsize = if (replace) nrow(x) else ceiling(0.632*nrow(x)),
  - nodesize = if (!is.null(y) && !is.factor(y)) 5 else 1,
  - maxnodes = NULL,
  - importance = FALSE, localImp = FALSE, nPerm = 1,
  - proximity, oob.prox = proximity,
  - norm.votes = TRUE, do.trace = FALSE,
  - keep.forest = !is.null(y) && is.null(xtest), corr.bias = FALSE,
  - keep.inbag = FALSE, …)

The residuals were calculated to compare the distinguishing performance of the two models by the “DALEX” package^61^. After determining the optimal machine learning model, the importance score of four m6A regulators was also evaluated using the “randomForest” package. “ggplots” and “pROC”^62^ packages were applied to visualize the results.

### Identification and evaluation of nomogram

These four key m6A-related genes were used to construct a nomogram using “rms”, and “rmda” R packages (https://CRAN.R-project.org/package=rmda). To estimate the performance of our classification and diagnostic models, we established a calibration curve, area under the receiver operating characteristic (AUC of ROC) curve, and risk decision curve analysis (DCA).

### Differences in immune characteristics and correlation analysis

The “CIBERSORT”^63^algorithm was utilized to calculate the levels of each immune cell infiltration of each sample based on the mRNA expression matrix, and “ggplot2” packages were utilized to reflect the difference of infiltrating immune cells between IS and control samples via violin diagram.

ssGSEA (single-sample gene-set enrichment analysis) algorithm was also applied to quantify the relative abundance of peripheral immune cell in IS using “GSVA” R package^64^. The Kruskal–Wallis test was performed to compare the difference between various samples. The gene set for marking each immune cell type was shown in Supplementary Table S5.

### Determination of the m6A modification pattern

Unsupervised clustering analysis was conducted to identify distinct m6A modification patterns in IS based on the expression of m6A regulators. The R package “ConsensuClusterPlus”(master.bioconductor.org/packages/release/bioc/html/ConsensusClusterPlus.html) implemented the above steps for 1000 iterations for guaranteeing the robustness of classification. We used the clustering score of the cumulative distribution function (CDF) curve to estimate the optimal number of clusters. A PCA analysis was performed to verify the reliability of consensus clustering. The m6A modulator expression, infiltrating immunocyte abundance score among the two distinct modification patterns were compared using “limma” packages, and “pheatmap”, “reshape2”, and “ggpubr” packages were utilized to visualize the results.

### Identification of differentially expressed genes (DEGs) between m6A distinct modification patterns

The empirical Bayesian approach of the “limma” R package was applied to screened DEGs between two m6A modification patterns. The significance criteria for determining DEGs were set as adjusted *p* < 0.05, |log2fold change (FC)|> 0.58. The common m6A regulator-mediated DEGs were overlapped by the Venn plot using the “VennDiagram” package (https://CRAN.R-project.org/package=VennDiagram). Additionally, Gene Ontology (GO) and Kyoto Encyclopedia of Genes and Genomes (KEGG) enrichment analyses^65–67^ were performed to explore the biological function of the six DEGs using the “clusterProfiler” R package (master.bioconductor.org/packages/release/bioc/html/clusterProfiler.html). The method of clustering the m6A regulators-related DEGs was similar to the way to identify the m6A modification patterns. The expression of m6A regulators associated genes was visualized by “enrichplot” (https://bioconductor.org/packages/enrichplot/),

“ComplexHeatmap” (Bioconductor—ComplexHeatmap) and “ggplot2” R packages.

### Generation of m6A related signature

We established a scoring system, m6Ascore, to assess the m6A modification features of each patient with IS. We then defined the m6Ascore using a method as the following formula: m6Ascore = $\sum ({\text{PC}}1i+{\text{PC}}2i)$, where i is the expression of m6 A phenotype-related genes described above. An alluvial diagram was used to visualize the attribute changes of an individual patient using the “gg alluvial” package in R (Alluvial Plots in ggplot2 • ggalluvial (corybrunson.github.io)).

### Expression and diagnostic performance of key m6A phenotype-related genes in IS

To identify the diagnostic performance of the six m6A phenotype-related genes, we first assessed the expression of six genes by the “limma” package, and “ggpubr” and “pheatmap” packages were utilized to display these results. In addition, we accessed the diagnostic performance of key m6A phenotype related-genes in the training (GSE16561) and validation datasets (GSE102541 and GSE140275) using the “pROC” R package. The area under the ROC curve (AUC of ROC), and 95% confidence interval (CI) were used to assess the discriminative power of a single gene or gene combination to distinguish IS from healthy.

#### Expression of neutrophil chemotaxis

The expression of neutrophil chemotaxis in different m6A patterns and genetic patterns was evaluated by the Wilcox test using the “limma” package in R. The significant threshold was set at *p* < 0.05.

#### Experimental validation

In terms of experimental validation, a total of 5 pairs of healthy and IS patients’ blood samples were collected at our hospital from June 2022 to November 2022. Informed consent was obtained from each patient, and the study was approved by the Ethics Committee of the Second affiliated hospital of Xi’an Jiaotong University. Total RNA was extracted and purified from collected blood samples using TRizol (Life Technologies). The reverse transcriptase kit (TaKaRa) was used to reverse total RNA into cDNA. qRT-PCR was performed on the Bio-Rad CFX system (Bio-Rad, Hercules, CA, USA) using SYBR Green Master Mix (TaKaRa). All gene expressions were normalized based on the β-actin mRNA levels in each sample using the 2 − ∆∆Ct method. The primer sequences were listed in Supplementary Table S6.

#### Statistics

R software (v4.2.1, https://www.r-project.org/) and GraphPad Prism software (GraphPad graphpad-prism.cn) for Windows (v9.0, San Diego, California, USA) were used to conduct all statistical analyses. The cut-off thresholds of differential analysis were set at *p* < 0.05 and |logFC|> 0.58. The experimental data were presented as mean ± SEM. For normally distributed variables, Student's t-test was used to compare the differences between the two groups, while the Mann–Whitney U test was used for abnormally distributed variables. *p* < 0.05 was considered a significant difference.

### Ethics approval and consent to participate

The datasets in this work were acquired from the publicly available datasets whose informed consent of patients were completed. Informed Consent were obtained from the study participants. Collecting and processing of human blood samples was in accordance with the Declaration of Helsinki and have been approved by Ethics Committee of the Second Affiliated Hospital of Xi'an Jiaotong University (No. 2019-218).

## Conclusion

In conclusion, this work demonstrated the extensive regulation mechanisms of m6A methylation modification in IS microenvironment. The difference in m6A modification patterns was an important factor that contributed to the formation of the heterogeneity and complexity of individual IS microenvironments. And m6A-related genes could be diagnostic biomarkers to identify IS patients which meanwhile might be beneficial to guide more effective immunotherapy strategies.

### Supplementary Information

Supplementary Information 1.

Supplementary Information 2.

Supplementary Information 3.

## Data Availability

All data used in this work can be acquired from the Gene-Expression Omnibus (GEO; (https://www.ncbi.nlm.nih.gov/geo; GSE16561, GSE102541, GSE140275). Experimental data was available in Supplementary materials.

## Abbreviations

AUC: Area under the curve
BBB: Blood–brain barrier
CDF: Consensus clustering cumulative distribution function
DCA: Decision curve analysis
DEGs: Differential expressed genes
GEO: Gene expression omnibus
GO: Gene ontology
HC: Healthy controls
IS: Ischemic stroke
KEGG: Kyoto encyclopedia of genes and genomes
m6A: N6-methyladenosine
PPI: Protein–protein interactions
RF: Random forest
ROC: Feceiver operating characteristic curve
SVM: Support vector machine
