# Supplementary material for: Identification of significant m6A regulators and immune microenvironment characterization in ischemic stroke
Source: Sci Rep. 2024 Feb 11;14:3456. doi: 10.1038/s41598-024-53788-5 (PMC10859379; doi:10.1038/s41598-024-53788-5)
Supplement: Supplementary file 3 — Supplementary Information 3. [file 41598_2024_53788_MOESM3_ESM.docx]

**Table S1. M6A clusters for IS samples**

| ID | METTL3 | RBM15B | YTHDF3 | RBMX | m6Acluster |
| --- | --- | --- | --- | --- | --- |
| GSM416528_treat | 2.023847 | 2.019582 | 1.703488 | 1.942541 | A |
| GSM416529_treat | 1.867365 | 2.049565 | 1.869825 | 1.844445 | A |
| GSM416530_treat | 1.779495 | 1.99873 | 1.766754 | 1.921109 | A |
| GSM416531_treat | 1.816688 | 1.975465 | 1.962156 | 1.90746 | B |
| GSM416532_treat | 1.974797 | 2.050407 | 2.124614 | 1.867271 | B |
| GSM416533_treat | 2.079521 | 1.873901 | 2.168995 | 2.018574 | B |
| GSM416534_treat | 1.905982 | 1.984818 | 2.176862 | 2.051936 | B |
| GSM416535_treat | 1.858634 | 1.847101 | 2.11361 | 1.930038 | B |
| GSM416536_treat | 1.977882 | 2.103577 | 2.112497 | 2.024111 | B |
| GSM416537_treat | 1.998673 | 2.055462 | 2.17967 | 1.712395 | B |
| GSM416538_treat | 1.83084 | 1.980282 | 1.855324 | 1.766754 | A |
| GSM416539_treat | 1.854132 | 2.010307 | 2.194117 | 1.899911 | B |
| GSM416540_treat | 2.014508 | 1.989014 | 2.149526 | 1.903961 | B |
| GSM416541_treat | 1.911543 | 2.050091 | 2.122364 | 1.940285 | B |
| GSM416542_treat | 2.063371 | 1.977822 | 2.169862 | 1.985171 | B |
| GSM416543_treat | 1.951685 | 2.023476 | 2.013703 | 1.925087 | B |
| GSM416544_treat | 2.060999 | 1.983009 | 2.133842 | 1.99751 | B |
| GSM416545_treat | 2.008574 | 1.921037 | 1.822861 | 2.042129 | A |
| GSM416546_treat | 2.018483 | 1.945647 | 1.956672 | 1.977983 | A |
| GSM416547_treat | 2.127126 | 1.948989 | 1.85177 | 1.962156 | A |
| GSM416548_treat | 1.929185 | 2.019532 | 1.834661 | 2.117727 | A |
| GSM416549_treat | 2.243677 | 1.985171 | 1.964864 | 2.110491 | A |
| GSM416550_treat | 1.89034 | 1.994526 | 1.890825 | 1.964223 | A |
| GSM416551_treat | 2.025698 | 1.949424 | 1.883775 | 2.009843 | A |
| GSM416552_treat | 2.192363 | 2.028101 | 1.964453 | 2.253061 | A |
| GSM416553_treat | 1.911021 | 1.921241 | 1.892354 | 2.026402 | A |
| GSM416554_treat | 1.930727 | 1.958247 | 1.965191 | 1.933911 | A |
| GSM416555_treat | 1.834341 | 2.023906 | 2.142006 | 1.860565 | B |
| GSM416556_treat | 1.967494 | 1.822422 | 2.012863 | 1.895756 | A |
| GSM416557_treat | 1.963084 | 1.946037 | 2.043876 | 2.004405 | B |
| GSM416558_treat | 1.842272 | 2.040448 | 1.914392 | 1.893442 | B |
| GSM416559_treat | 2.001179 | 1.919341 | 2.111425 | 2.05968 | B |
| GSM416560_treat | 1.822861 | 1.946355 | 2.16217 | 1.977606 | B |
| GSM416561_treat | 1.893442 | 2.100331 | 1.97486 | 1.831852 | B |
| GSM416562_treat | 2.063371 | 1.96679 | 2.213497 | 2.019164 | B |
| GSM416563_treat | 1.900076 | 2.015705 | 2.23193 | 1.783682 | B |
| GSM416564_treat | 1.792698 | 1.997728 | 2.174705 | 1.880149 | B |
| GSM416565_treat | 2.04768 | 1.913717 | 1.937127 | 1.971378 | A |
| GSM416566_treat | 1.916911 | 2.0141 | 2.02855 | 2.080926 | B |

**Table S2. m6A regulators associated gene clusters for IS samples**

| ID | geneCluster |
| --- | --- |
| GSM416528_treat | A |
| GSM416529_treat | A |
| GSM416530_treat | A |
| GSM416531_treat | A |
| GSM416532_treat | B |
| GSM416533_treat | B |
| GSM416534_treat | B |
| GSM416535_treat | B |
| GSM416536_treat | B |
| GSM416537_treat | B |
| GSM416538_treat | B |
| GSM416539_treat | B |
| GSM416540_treat | B |
| GSM416541_treat | B |
| GSM416542_treat | B |
| GSM416543_treat | B |
| GSM416544_treat | B |
| GSM416545_treat | A |
| GSM416546_treat | A |
| GSM416547_treat | A |
| GSM416548_treat | A |
| GSM416549_treat | A |
| GSM416550_treat | A |
| GSM416551_treat | A |
| GSM416552_treat | A |
| GSM416553_treat | A |
| GSM416554_treat | A |
| GSM416555_treat | B |
| GSM416556_treat | B |
| GSM416557_treat | B |
| GSM416558_treat | B |
| GSM416559_treat | B |
| GSM416560_treat | B |
| GSM416561_treat | B |
| GSM416562_treat | B |
| GSM416563_treat | B |
| GSM416564_treat | B |
| GSM416565_treat | B |
| GSM416566_treat | B |

**Table S4. m6A regulators investigated in the study**

| Gene | Type |
| --- | --- |
| METTL3 | writers |
| METTL14 | writers |
| METTL16 | writers |
| WTAP | writers |
| VIRMA | writers |
| ZC3H13 | writers |
| RBM15 | writers |
| RBM15B | writers |
| CBLL1 | writers |
| YTHDC1 | readers |
| YTHDC2 | readers |
| YTHDF1 | readers |
| YTHDF2 | readers |
| YTHDF3 | readers |
| HNRNPC | readers |
| FMR1 | readers |
| LRPPRC | readers |
| HNRNPA2B1 | readers |
| IGFBP1 | readers |
| IGFBP2 | readers |
| IGFBP3 | readers |
| RBMX | readers |
| ELAVL1 | readers |
| IGF2BP1 | readers |
| FTO | erasers |
| ALKBH5 | erasers |

**Table S6. Sequences of primers**

| Primer name | Sequence |
| --- | --- |
| YTHDF3-F | 5’-GCTACTTTCAAGCATACCACCTC-3’ |
| YTHDF3-R | 5’-ACAGGACATCTTCATACGGTTATTG-3’ |
| METTL3-F | 5’-CTATCTCCTGGCACTCGCAAGA-3’ |
| METTL3-R | 5’-GCTTGAACCGTGCAACCACATC-3’ |
| RBM15B-F  RBM15B-R  RBMX-F  RBMX-R | 5’-TGGTAACCTGGACCACAGCGTA-3’  5’-GGTTCTGGAACTTGAGGAAGGC-3’  5’-AGACGCTAAGGATGCAGCCAGA-3’  5’-CCACCTCTAAGACCTCTTGGAG-3’ |
| APOBEC3A-F | 5’-GACAATGGCACCTCGGTCAAGA-3’ |
| APOBEC3A-R | 5’-CCAACTGCAAAGAAGGAACCAGG-3’ |
| FCGR3A-F | 5’-GGTGACTTGTCCACTCCAGTGT-3’ |
| FCGR3A-R | 5’-ACCATTGAGGCTCCAGGAACAC-3’ |
| FTHL11-F | 5’-GCTGAATGCGATGGAGTGTGCA-3’ |
| FTHL11-R | 5’-GGCTTTCACCTGCTCATTCAGG-3’ |
